# Inhibition of Aurora-B suppresses HepG2 cell invasion and migration via the PI3K/Akt/NF-κB signaling pathway *in vitro*

**DOI:** 10.3892/etm.2014.1844

**Published:** 2014-07-14

**Authors:** REN FENG SHAN, YUN FEI ZHOU, AI FEN PENG, ZHI GANG JIE

**Affiliations:** 1 Department of General Surgery, First Affiliated Hospital of Nanchang University, Nanchang, Jiangxi 330006, P.R. China; 2 Department of Orthopedics, First Affiliated Hospital of Nanchang University, Nanchang, Jiangxi 330006, P.R. China; 3 College of Humanity, Jiangxi University of Traditional Chinese Medicine, Nanchang, Jiangxi 330006, P.R. China

**Keywords:** Aurora-B, HepG2, invasion, migration, phosphoinositide 3-kinase/Akt/nuclear factor-κB signaling pathway

## Abstract

In the present study, the effect of Aurora-B inhibition on HepG2 cell invasion and migration *in vitro* was investigated. A recombinant plasmid targeting the Aurora-B gene (MiR-Aurora-B) was used to inhibit Aurora-B expression in HepG2 cells. Cell migration and invasion were investigated using Transwell migration and invasion assays. The results demonstrated that cell invasion and migration were suppressed by inhibiting Aurora-B. In addition, the effect of Aurora-B inhibition on the activity of the phosphoinositide 3-kinase (PI3K)/Akt/nuclear factor (NF)-κB signaling pathway was investigated by analyzing the protein expression levels of phosphorylated (p)-Akt, Akt, NF-κB p65, matrix metalloproteinase (MMP)-2 and MMP-9 using western blot analysis. The results demonstrated that the protein expression levels of p-Akt, NF-κB p65, MMP-2 and MMP-9 were reduced significantly by inhibiting Aurora-B. Therefore, inhibition of Aurora-B was shown to suppress hepatocellular carcinoma cell migration and invasion by decreasing the activity of the PI3K/Akt/NF-κB signaling pathway *in vitro*.

## Introduction

Hepatocellular carcinoma (HCC) is the fifth most common malignancy worldwide (1,2). Although surveillance of patients with risk factors for HCC and the development of locoregional treatment options have improved outcomes, to date, there are no effective curative methods due to the high invasion, early metastasis and high tumor recurrence rates of HCC following surgery or interventional treatment. Furthermore, HCC progression, including metastasis, contributes to the high fatality rates of liver cancer. Lymph node metastasis of tumors is considered to be an important factor involved in HCC progression. However, the underlying molecular mechanisms involved in lymph node metastasis of HCC remain unclear.

Aurora kinases are key regulators of protein phosphorylation during mitosis (3), consisting of three members that differ with regard to subcellular localization, activation kinetics and function. Aurora-B is one of the major protein kinases that ensures the proper execution and fidelity of mitosis. As a member of the chromosomal passenger complex (CPC), Aurora-B has been implicated in various mitotic functions, including chromosome-microtubule interactions, sister chromatid cohesion, the spindle-assembly checkpoint and cytokinesis. Accumulating evidence indicates that Aurora-B is an important antitumor target that is strongly associated with lymph node metastasis in various tumor types (4–7). Previous studies have demonstrated that the expression of Aurora-B is increased in HCC cells, and the inhibition of Aurora-B suppresses cell proliferation and invasion *in vitro* and *in vivo* (8–11). However, the potential molecular mechanisms underlying the involvement of Aurora-B in HCC development and lymph node metastasis remain unclear.

Matrix metalloproteinases (MMPs) are involved in the degradation of the basement membrane and epimatrix, among which MMP-2 and -9 markedly correlate with tumor invasion. A number of studies have revealed that activation of the gene encoding nuclear factor (NF)-κB, the upstream regulator of MMPs, promotes tumor cell invasion and migration (12,13). In addition, phosphorylation and activation of Akt has been recognized as an important regulatory factor in the NF-κB signaling pathway. A previous study demonstrated that an inhibitor of Aurora-B decreased Akt phosphorylation at Ser 473 (14). Therefore, we hypothesized that the inhibition of Aurora-B results in the suppression of HCC cell invasion and migration via decreasing the activity of the phosphoinositide 3-kinase (PI3K)/Akt/NF-κB signaling pathway.

Thus, the aim of the present study was to investigate whether the inhibition of Aurora-B caused the suppression of HepG2 cell invasion and migration via decreasing the activity of the PI3K/Akt/NF-κB signaling pathway *in vitro*.

## Materials and methods

### Construction of the recombinant plasmid containing miRNA targeting the Aurora-B gene

A human cDNA sequence encoding the Aurora-B protein was obtained from GenBank (NM-004217), and single-stranded DNA oligos were designed and synthesized using the following primer sequences: Forward, 5′-CCGGGAAGAGCTGCACATTTGACGACTCGAGTCGTCAAATGTGCAGCTCTTCTTTTTG-3′ and reverse, 5′-AATTCAAAAAGAAGAGCTGCACATTTGACGACTCGAGTCGTCAAATGTGCAGCTCTTC-3′.The products were cloned into the express vector, pcDNA6.2-GW/EmGFP-miR, using a BLOCK-iT™ Pol II miR RNAi Expression Vector kit with EmGFP (K4936-00; Invitrogen Life Technologies, Carlsbad, CA, USA). The DNA sequence of the plasmid was confirmed using a PureLink HiPure Plasmid DNA kit (K2100-03; Invitrogen Life Technologies).

### Cell culture and transfection

A human HCC cell line, HepG2, (Shanghai Cell Bank of the Chinese Academy of Sciences, Shanghai, China) was cultured in Dulbecco’s modified Eagle’s medium (DMEM) supplemented with 10% fetal bovine serum (FBS) and incubated at 37°C in 5% CO_2_. The HepG2 cells were seeded in six-well plates at 30% confluence on the day prior to transfection. Transfection with the recombinant plasmid targeting the Aurora-B gene (MiR-Aurora-B) or the negative plasmid (MiR-Neg) was performed using Lipofectamine 2000 (Invitrogen Life Technologies). Transfection complexes were prepared according to the manufacturer’s instructions.

### Quantitative polymerase chain reaction (qPCR)

The qPCR procedure was performed as follows: 95°C for 2 min; 35 cycles of 95°C for 20 sec, 55°C for 30 sec and 72°C for 30 sec; and annealing between 65 and 95°C with 0.5°C progressive increases. The primers used in the study were as follows: Aurora-B (NM-004217) forward, 5′-ATAGCAGTGGGACACCCGACAT-3′ and reverse, 5′-GGGACTTGAAGAGGACCTTGAGC-3′; and glyceraldehyde 3-phosphate dehydrogenase (GAPDH; NM-002046) forward, 5′-CCTGTTCGACAGTCAGCCGCATC-3′ and reverse, 5′-CGACCAAATCCGTTGACTCCGACC-3′.

### Western blot analysis

Total protein from the cells was extracted using radioimmunoprecipitation assay lysis buffer containing 60 μg/ml phenylmethylsulfonyl fluoride. Protein concentration was determined using a Bradford assay. Equal amounts of protein were electrophoresed using 8% SDS-PAGE and transferred onto a pure nitrocellulose blotting membrane (0.22 μM). The membranes were blocked with 5% skimmed milk for 1 h at room temperature, then incubated with primary antibodies [rabbit anti-Aurora-B immunoglobulin G (IgG), 1:200; rabbit anti-phosphorylated (p)-Akt IgG, 1:800; goat anti-Akt IgG, 1:1,000; rabbit anti-NF-κB, 1:500; and rabbit anti-MMP-2 and MMP-9, 1:1,000] overnight at 4°C. The membranes were washed prior to incubation with the appropriate horseradish peroxidase (HRP)-conjugated secondary antibodies (anti-rabbit, -goat and -mouse, 1:2,000). The immune complexes were detected with a pro-light HRP kit (Tiangen Biotech Co., Ltd., Beijing, China). GAPDH (1:1,000; Cell Signaling Technology, Inc., Danvers, MA, USA) protein expression was used as a normalization control for protein loading. All the experiments were repeated six times over multiple days.

### Transwell invasion and migration assays

Cell invasion was assayed using a Transwell chamber (Millipore Corporation, Billerica, MA, USA) with Matrigel (BD Biosciences, Franklin Lakes, NJ, USA). For the invasion assay, a Transwell chamber was placed in a six-well plate and coated with 30 μl Matrigel, which was incubated for 40 min at 37°C. In the two Transwell assays, 24 h following transfection, the cells were trypsinized and seeded in chambers at a density of 5×10^4^ cells/well and cultured in DMEM with 2% serum, while 600 μl FBS-DMEM (10%) was added to the lower chamber. After 24 h, the migrated cells were fixed with 100% methanol for 30 min. The non-migrated cells were removed with cotton swabs. Next, the cells on the bottom surface of the membrane were stained with crystal violet for 20 min. Cell images were obtained under a phase-contrast microscope (Olympus, Tokyo, Japan), and cell counts were performed using Image J software (National Institutes of Health, Bethesda, MD, USA). Cell migration was assessed by determining the ability of the cells to move into a cellular space in a two-dimensional *in vitro* wound healing assay’ In brief, cells were grown to confluence in 6-well tissue culture plastic dishes to a density of ~5 × 10^6^ cells/well. Cells were detached by dragging a rubber policeman (Fisher Scientific, Hampton, NH, USA) through the center of the plate. Cultures were rinsed with PBS and replaced with fresh DMEM or DMEM containing 10% FBS, after which the cells were incubated at 37°C for 24 h. Images were captured at 0 and 24 h and the migrated distance was measured using ImageJ (NIH, Bethesda, MD, USA). Transwell invasion and migration assays were repeated six times over multiple days.

### Statistical analysis

All measurement data are presented as the mean ± standard deviation. Statistical analysis was performed using a t-test for two independent-samples, where P<0.05 was considered to indicate a statistically significant difference. All analyses were performed using SPSS version 13.0 (SPSS, Inc., Chicago, IL, USA).

## Results

### Effect of the recombinant plasmid targeting the Aurora-B gene on Aurora-B expression in HepG2 cells

**Figure 1:**
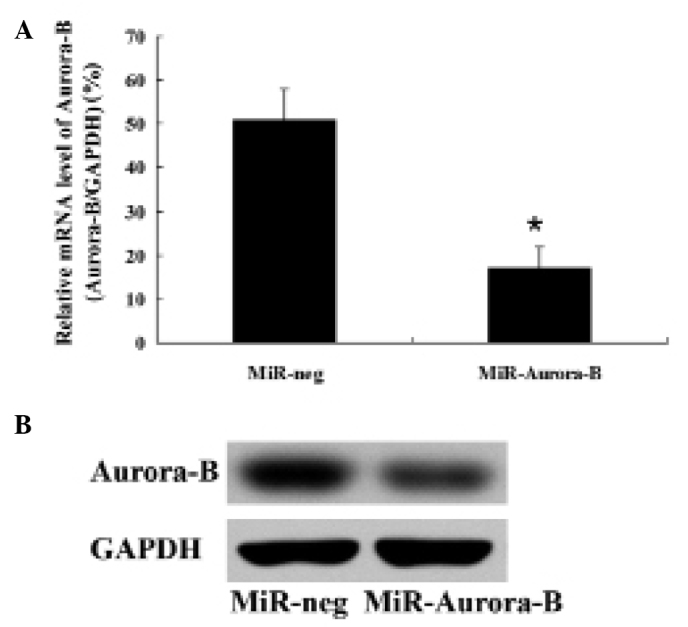
Effect of the recombinant plasmid targeting the Aurora-B gene on Aurora-B expression in HepG2 cells. (A) Analysis of the mRNA expression level of Aurora-B in HepG2 cells by quantitative polymerase chain reaction. The relative mRNA expression level of Aurora-B (Aurora-B/GAPDH) in the cells transfected with the recombinant plasmid was significantly lower compared with those transfected with the negative control plasmid. Data are presented as the mean ± standard deviation (n=6). ^*^P<0.05, vs. negative control. (B) Analysis of Aurora-B protein expression levels in HepG2 cells by western blot analysis. Aurora-B protein expression in the cells transfected with the recombinant plasmid was significantly lower compared with those transfected with the negative control plasmid. GADPH, glyceraldehyde 3-phosphate dehydrogenase.

Cultured HepG2 cells were transfected with the recombinant plasmid for 6 h and then cultured for 48 h. Aurora-B mRNA and protein expression levels in the HepG2 cells were detected by qPCR and western blot analysis, respectively. Aurora-B mRNA and protein expression levels in the cells transfected with the recombinant plasmid were significantly lower compared with those transfected with the negative control plasmid (Fig. 1). These observations indicated that the recombinant plasmid miRNA targeting the Aurora-B gene decreased Aurora-B expression in HepG2 cells.

### Inhibition of Aurora-B suppresses HepG2 cell invasion and migration in vitro

**Figure 2:**
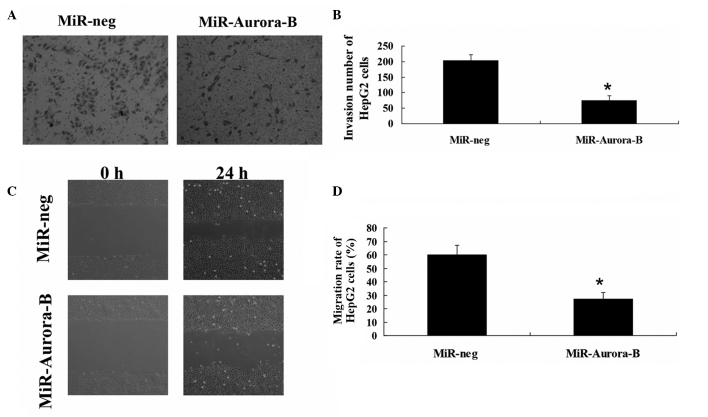
Inhibiting Aurora-B suppresses HepG2 cell invasion and migration *in vitro*. (A and B) Analysis of the invasion ability of HepG2 cells using a Transwell invasion assay. The invasion rate was significantly lower in the cells transfected with the recombinant plasmids compared with the negative control. Data are presented as the mean ± SD (n=6). ^*^P<0.05, vs. negative control group. (C and D) Analysis of the migration ability of HepG2 cells using a wound healing assay. The migration rate was significantly lower in the cells transfected with the recombinant plasmids compared with the negative control. Data are presented as the mean ± SD (n=6). ^*^P<0.05, vs. negative control group. GADPH, glyceraldehyde 3-phosphate dehydrogenase; SD, standard deviation.

HepG2 cells were transfected with MiR-Aurora-B or MiR-Neg. Transwell invasion and wound healing assays were performed to investigate the migration and invasion of HepG2 cells. The results revealed that the rates of invasion and migration in the cells transfected with MiR-Aurora-B were significantly lower compared with those transfected with MiR-Neg (Fig. 2). These results indicated that Aurora-B inhibition was able to suppress HepG2 cell invasion and migration *in vitro*.

### Inhibition of Aurora-B decreases the activity of the PI3K/Akt/NF-κB signaling pathway

**Figure 3:**
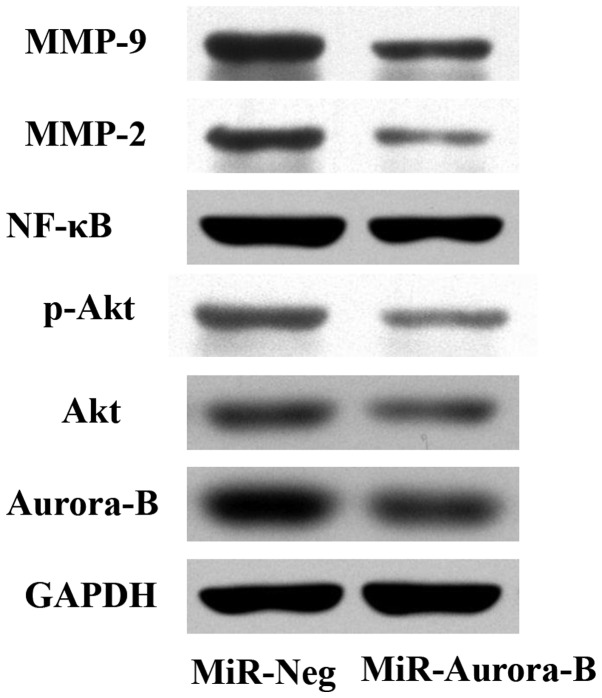
Inhibiting Aurora-B expression decreases the activity of the PI3K/Akt/NF-κB signaling pathway. With the downregulation of Aurora-B, the p-Akt, Akt, MMP-2, MMP-9 and NF-κB protein expression levels decreased significantly in the cells transfected with MiR-Aurora-B when compared with the cells transfected with MiR-Neg. NF-κB, nuclear factor-κB, MMP, matrix metalloproteinase; PI3K, phosphoinositide 3-kinase,

To investigate the effect of Aurora-B inhibition on the phosphorylation of Akt, the protein expression level of p-Akt was analyzed in HepG2 cells using western blot analysis. The results demonstrated that the p-Akt protein expression level in the cells transfected with MiR-Aurora-B was significantly lower compared with the cells that had been transfected with MiR-Neg (Fig. 3). This observation indicated that the inhibition of Aurora-B may decrease the phosphorylation of Akt. In addition, the protein expression levels of Akt, NF-κB p65, MMP-2 and MMP-9 were detected, and were shown to decrease significantly in the cells that had been transfected with MiR-Aurora-B, as compared with the cells transfected with MiR-Neg. These observations demonstrated that inhibition of Aurora-B gene expression downregulated MMP-2, MMP-9 and NF-κB protein expression levels in HepG2 cells (Fig. 3), indicating that Aurora-B inhibition decreases the activity of the PI3K/Akt/NF-κB signaling pathway.

## Discussion

Aurora kinases are serine/threonine kinases that are essential for cell cycle control and mitosis. Mammals have three Aurora kinase family members (A, B and C), and these kinases are expressed at maximum levels during mitosis. Aurora-B, part of the CPC, is located on the chromosome arms during prophase and at the centromeres during prometaphase and metaphase. Aurora-B subsequently localizes to the midbody during cytokinesis. Increased expression levels of Aurora-B have been identified in numerous types of cancer (15–17). Various studies have demonstrated that inhibition of Aurora-B inhibits cell proliferation and induces cell apoptosis in a variety of tumor types (17–19). These observations have led to an interest in Aurora-B as a molecular target for cancer treatment. Notably, recent studies have shown that upregulation of Aurora-B expression is associated with tumor cell metastasis (5), while downregulation of Aurora-B expression inhibits cell invasion and migration in a number of tumor types (19,20). A previous study revealed that inhibition of Aurora-B using a small molecular inhibitor suppressed the growth of HCC cells (11). However, the effects of Aurora-B inhibition on HCC cell invasion and migration are yet to be fully elucidated. Therefore, the aim of the present study was to investigate the effect of Aurora-B inhibition on HCC cell migration and invasion. A plasmid targeting Aurora-B was used to inhibit Aurora-B expression in HepG2 cells, and the rates of HepG2 cell migration and invasion were investigated with Transwell assays. The results demonstrated that the rates of migration and invasion were significantly lower in the cells transfected with MiR-Aurora-B compared with those treated with MiR-Neg, indicating that Aurora-B inhibition suppresses HepG2 cell migration and invasion *in vitro*.

In addition, potential molecular mechanisms associated with Aurora-B inhibition of HCC cell migration and invasion were analyzed. The PI3K/Akt/NF-κB signaling pathway is known to be important in the metastasis of malignant tumors (21). Long *et al* demonstrated that inhibition of Aurora-B using a small molecular inhibitor was able to decrease the phosphorylation of Akt at Ser 473 (14). Phosphorylation of Akt is essential for NF-κB activation via the stimulation of the IκB kinase complex, which phosphorylates and inactivates IκB, an inhibitor of NF-κB. Previously, NF-κB was demonstrated to upregulate MMP-9 expression (22), while the inhibition of NF-κB was identified to downregulate MMP-2 expression (23). During the development of metastases, cancer cells degrade the components of the extracellular matrix. MMPs, particularly MMP-2 and -9, are associated with this process due to their capacity to degrade the extracellular matrix, promoting tumor invasion. In the present study, the protein expression levels of Akt, p-Akt, NF-κB p65, MMP-2 and MMP-9 were detected by western blot analysis to investigate whether Aurora-B inhibition resulted in the decreased activity of the PI3K/Akt/NF-κB signaling pathway. The results demonstrated that the protein expression levels of p-Akt, Akt, NF-κB p65, MMP-2 and MMP-9 were significantly decreased in the cells transfected with MiR-Aurora-B when compared with those transfected with MiR-Neg. These results indicate that inhibition of Aurora-B decreases the activity of the PI3K/Akt/NF-κB signaling pathway in HepG2 cells.

In conclusion, the observations of the present study indicate that inhibition of Aurora-B suppresses HepG2 cell invasion and migration via modulation of the PI3K/Akt/NF-κB signaling pathway *in vitro*. Thus, inhibitors targeting Aurora-B and the PI3K/Akt/NF-κB pathway may be potential therapeutic strategies for treating HCC metastases.
